# Supplementary material for: Longitudinal variation in resilient psychosocial functioning is associated with ongoing cortical myelination and functional reorganization during adolescence
Source: Nat Commun. 2024 Jul 29;15:6283. doi: 10.1038/s41467-024-50292-2 (PMC11286871; doi:10.1038/s41467-024-50292-2)
Supplement: Supplementary file 1 — Supplementary Information [file 41467_2024_50292_MOESM1_ESM.pdf]

Supplementary Materials

Longitudinal variation in resilient psychosocial functioning is associated with ongoing cortical myelination and functional reorganization during adolescence

Meike D. Hettwer, Lena Dorfschmidt, Lara M.C. Puhlmann, Linda M. Jacob, Casey Paquola, Richard A. Bethlehem, NSPN Consortium, Edward T. Bullmore, Simon B. Eickhoff, Sofie L. Valk

Contains:

Supplementary Results
Supplementary Methods
Supplementary Table S1-S3
Supplementary Figures S1-S11
NSPN Consortium author list
Supplementary References

Supplementary Results

Distribution of distress scores and resilient psychosocial functioning with respect to demographics

Having derived distress scores (see **Supplementary Table S1** for loadings) and resilient psychosocial functioning (Res_{PSF}) scores and their intra-individual rates of change (Δ), we tested whether scores differed between self-reported sexes or as an effect of age (see **Supplementary Table S2** for statistics). In the larger sample of 712 individuals, we observed significantly higher Res_{PSF} and lower distress levels in males than in females. This sex difference was not significant in the smaller sample (n = 141) included in the imaging analyses. Neither Res_{PSF} nor distress scores were significantly associated with age. Intra-individual change in Res_{PSF} and distress showed no sex or age effects in neither sub-sample. As change in Res_{PSF} was the main behavioral variable of interest in the current study and did not show a significant sex difference, we did not perform further sex-stratified analyses.

Table S1. Factor loadings of questionnaires included for the computation of a general distress factor.

Item	*Loading*
**ypq 84 no good at all**	0.803
**ypq 89 useless**	0.783
**ypq 92 failure**	0.782
**ypq 51 others happier**	0.769
**ypq 23 hated myself**	0.769
**ypq 08 no good any more**	0.767
**ypq 45 alone with people**	0.755
**ypq 30 never as good**	0.739
**ypq 31 everything wrong**	0.725
**ypq 38 worried lots**	0.725
**ypq 50 worried happen**	0.725
**ypq 59 worried**	0.724
**ypq 88 not proud**	0.716
**ypq 39 afraid lots**	0.712
**ypq 36 others do easily**	0.708
**ypq 27 lonely**	0.705
**ypq 15 no good future**	0.702
**ypq 16 not worth living**	0.691
**ypq 28 nobody loved me**	0.689
**ypq 44 what others think**	0.681
**ypq 22 bad things happen**	0.68
**ypq 61 worried bad**	0.675
**ypq 35 things went wrong**	0.674
**ypq 21 hard to think**	0.67
**ypq 25 looked ugly**	0.654
**ypq 53 hurt fussed**	0.649
**ypq 42 others dislike way**	0.647
**ypq 24 bad person**	0.644
**ypq 66 bad nothing wrong**	0.643
**ypq 72 unsure right things**	0.642
**ypq 60 against me**	0.639
**ypq 09 blame myself**	0.637
**ypq 54 did things wrong**	0.636
**ypq 56 worried night**	0.635
**ypq 20 not see friends**	0.622
**ypq 01 miserable**	0.613
**ypq 17 dying**	0.608
**ypq 02 not enjoy**	0.595
**ypq 18 family better off**	0.584
**ypq 19 kill myself**	0.581
**ypq 29 no fun**	0.576
**ypq 49 tired a lot**	0.575
**ypq 40 angry easily**	0.571
**ypq 91 more respect**	0.565
**ypq 12 talk less**	0.563
**ypq 11 grumpy**	0.558
**ypq 46 often sick**	0.557
**ypq 41 worry parents say**	0.549
**ypq 34 trouble making mind**	0.545
**ypq 69 something wrong**	0.541
**ypq 14 cried**	0.524
**ypq 43 hard to sleep**	0.517
**ypq 32 sleep badly**	0.504
**ypq 52 bad dreams**	0.486
**ypq 55 wake scared**	0.48

**ypq 05 tired**	0.479
**spq 09 talked about**	0.467
**ypq 63 over and over**	0.455
**ypq 06 moving slowly**	0.451
**ypq 58 wiggled seat**	0.436
**ypq 07 restless**	0.435
**ypq 62 certain things**	0.433
**spq 60 others watching**	0.42
**spq 63 people talk**	0.413
**ypq 13 talk slowly**	0.412
**ypq 67 clean enough**	0.397
**ypq 26 aches pains**	0.396
**ypq 37 getting breath**	0.392
**spq 64 hear thoughts**	0.374
**spq 61 distract sounds**	0.349
**ypq 64 hated dirt**	0.318
**ypq 73 broke rules**	0.315
**spq 04 mistake objects**	0.304
**ypq 78 cheated**	0.303
**spq 13 force around you**	0.291
**ypq 68 special way**	0.285
**ypq 70 hands clean**	0.281
**ypq 71 special words**	0.28
**spq 31 thoughts aloud**	0.264
**ypq 65 special number**	0.255
**spq 40 saw invisible**	0.245

**ypq 77 skived**	0.244
**spq 28 special sign**	0.231
**ypq 79 ran away**	0.231
**ypq 76 hurt someone**	0.218
**ypq 74 stole**	0.21
**ypq 75 damage property**	0.163
**ypq 83 hurt animal**	0.133
**wemwbs 05 energy**	-0.418
**wemwbs 13 interested**	-0.454
**wemwbs 09 feel close**	-0.507
**wemwbs 11 make mind**	-0.549
**wemwbs 12 loved**	-0.553
**wemwbs 01 optimistic**	-0.559
**ypq 90 as good**	-0.574
**ypq 86 good qualities**	-0.575
**wemwbs 02 useful**	-0.581
**wemwbs 03 relaxed**	-0.582
**wemwbs 06 problems**	-0.598
**ypq 87 do things well**	-0.608
**ypq 85 satisfied**	-0.61
**ypq 93 positive**	-0.64
**wemwbs 07 think clear**	-0.645
**wemwbs 10 confident**	-0.669
**wemwbs 14 cheerful**	-0.702
**wemwbs 08 feel good**	-0.713

Ypg = Young person's questionnaire (containing: Moods and Feelings Questionnaire, Revised Children's Manifest Anxiety Scales, Leyton Obsessional Inventory, The Behaviours Checklist, Rosenberg Self-Esteem Scale); spq = Schizotypal Personality Questionnaire; wemwbs = Warwick-Edinburgh Mental Wellbeing Scale.

Table S2. Distribution of distress and resilient psychosocial functioning (Res_{PSF}) scores with respect to sex (mean +/- SD) and age in the prediction sample (i.e., 712 individuals included for the computation of Res_{PSF}; 455 with repeated measures) and the imaging sample (i.e., 141 individuals for which both imaging and behavioral data was available for two time points).

	Prediction sample (n = 712)	***Imaging sub-sample (n = 141)***
Mean Res_{PSF}	Male: 2.46 ± 16.93<br>Female: -2.74 ± 19.36<br>**t(710) = -3.81, p = 0.0001, [-7.89, -2.52]**<br><br>Correlation with age: r = 0.04, p = 0.22	Male: 3.59 ± 17.08<br>Female: 0.83 ± 16.16<br>t(139) = -0.99, p = 0.32, CI = [-8.3 2.77]<br><br>Correlation with age: r = 0.08, p = 0.32
ΔRes_{PSF}	Male: 0.60 ± 15.84<br>Female: 1.97 ± 10.84<br>t(453) = 0.81, p = 0.42, CI = [-1.98, 4.73]<br><br>Correlation with age: -0.06, p = 0.20	Male: 2.62 ± 15.83<br>Female: 2.18 ± 16.96<br>t(139) = -0.16, p = 0.88, CI = [-5.9, 5,02]<br><br>Correlation with age: r = -0.01, p = 0.9
Mean distress	Male: -6.39 ± 20.07<br>Female: -1.43 ± 21.43<br>**t(710) = 3.15, p = 0.002, CI = [1.85, 7.97]**<br><br>Correlation with age: r = 0.08, p = 0.04	Male: -8.70 ± 20.70<br>Female: -5.91 ± 18.14<br>t(139) = 0.85, p = 0.39, CI = [-3.67, 9.27]<br><br>Correlation with age: r = 0.01, p = 0.86
Δdistress	Male:-3.15 ± 16.03<br>Female: -5.12 ± 20.44<br>t(453) = -0.90, p = 0.37, CI = [-5.01, 1.87]<br><br>Correlation with age: r = 0.03, p = 0.57	Male: -0.91 ± 16.77<br>Female: 2.15 ± 17.64<br>t(139) = 1.06, p = 0.29, CI = [-2.67, 8.80]<br><br>Correlation with age: r = 0.05, p = 0.22

Res_{PSF} = Resilient psychosocial functioning scores. Bold = significant at p<0.05.

Potential links between changes in Res_{PSF} and changes in adversity exposure

Given that Res_{PSF} were computed separately for each time point by predicting distress levels based on adversity levels measured at each time point, our approach inherently adjusts for changes in adversity exposure between measurement time points. We still wanted to confirm that changes in Res_{PSF} were not simply a correlate of increasing or decreasing adversity levels. Correlating ΔRes_{PSF} with Δs of individual risk assessment scores revealed no significant associations between ΔRes_{PSF} and Δ-scores from the MOPS ($r = 0.10$), APQ ($r = 0.10$), LEQ ($r = -0.02$; all $p > 0.05$), or SES (remained unchanged for most individuals; **Supplementary Figure S1**). Changes in adversity exposure were further not associated with changes in myelin-sensitive MT (all $p > 0.05$).

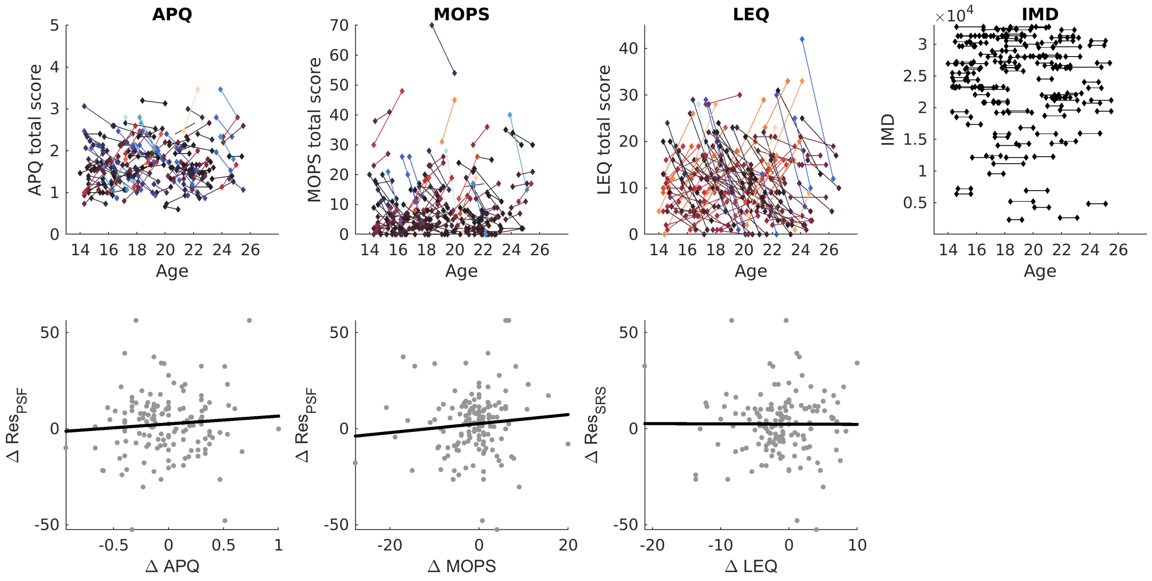

Supplementary Figure S1. ***Changes in adversity exposure between measurement timepoints.*** The upper row depicts intra-individual changes in adversity measures (n = 141 individuals). The lower row depicts respective Pearson's correlations between changes in adversity measures and changes in resilient psychosocial functioning (ΔRes_{PSF}; n=141). No significant association with ΔRes_{PSF} was observed for change in adversity exposure as captured by the Measure of Parenting Style (MOPS; $r = 0.10$, $p = 0.24$), the Alabama parenting questionnaire (APQ; $r = 0.10$, $p = 0.21$), or the Life events questionnaire (LEQ; $r = -0.02$, $p = 0.79$). IMD = Index of mean deprivation.

Representativeness of the MRI subsample

To address concerns that developmental neuroimaging studies may not be representative of the general developmental population [1], we descriptively compared the distributions of levels of environmental risk exposures as well as behavioral outcome measures in n = 144 individuals included in neuroimaging analyses (the imaging subsample) and the larger sample of individuals included in behavioral analyses only (the non-imaging subsample; **Supplementary Figure S2**). We generally observed strongly overlapping distributions for distress and resilient psychosocial functioning (Res_{PSF}) scores, as well as for APQ, MOPS, LEQ and CTQ questionnaires, between the two sub-samples. For SES, considered here as an index of mean deprivation (IMD), we observed a comparable range but a relatively higher proportion of higher SES in the imaging sub-sample (i.e., a more left-skewed distribution). This suggests a potential oversampling of individuals from higher socioeconomic backgrounds for the neuroimaging analysis. At the same time, it should be noted that the behavioral outcome measure (Res_{PSF}) was computed in the full sample, including more individuals with lower SES, and also that SES was weighted with the lowest feature importance by the model used to predict Res_{PSF} (**Figure 1A**).

We also note that the NSPN sample is a locally collected sample from London and Cambridgeshire in the UK. 75% of the total sample and 84% of the imaging sub-sample were white. Moreover, the sample is predominantly healthy, thereby excluding individuals with, for example, neurodevelopmental disorders who are part of the general population. It will be important to test the replicability of the current results in more socio-economically and ethnically representative samples of the general population. The efficacy of potential resilience factors implied by the current study in a largely white, relatively affluent and healthy sample requires further validation in ethnically, socio-economically and clinically defined groups that are underrepresented in this sample.

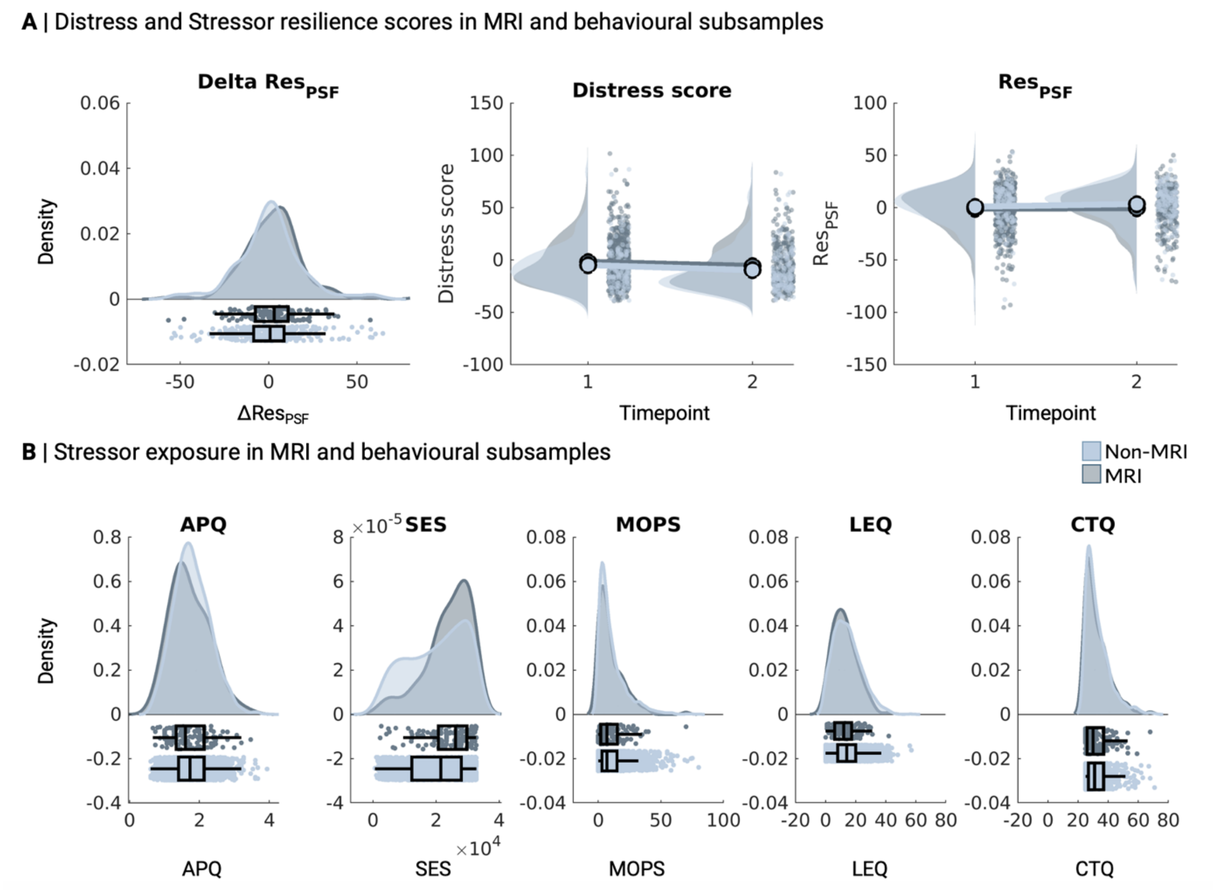

Supplementary Figure S2. ***Representativeness of the MRI subsample.*** The MRI subsample comprises the n = 141 individuals included in the main analyses linking changes in resilient psychosocial functioning (Res_{PSF}) scores to myeloarchitectonic and functional maturation. The Non-MRI sample comprises all other individuals that were included in behavioral analyses only and for whom respective behavioral data were available: In A), the non-MRI sub-sample includes n = 314 individuals with longitudinal ΔRes_{PSF} scores and n=885 individuals with distress scores. In B), the non-MRI sub-sample comprises n = 1457 individuals with all risk exposure assessments completed. Distributions show density plots. APQ = Alabama Parenting Questionnaire; MOPS = Measure of Parenting Style; LEQ = Life Events Questionnaire; IMD = Index of Mean Deprivation.

*Sensitivity tests for ΔRes$_{PSF}$ * ΔMT effects*

We tested whether the observed association between ΔRes$_{PSF}$ and ΔMT was robust to analytical choices by spatially correlating (Pearson's r) the original unthresholded t-map with t-maps derived from alternative analytical approaches (**Supplementary Figure S3A**). We observed virtually unchanged results when 1) not including mean Res$_{PSF}$ as a covariate in the general model (r = 1 with the original map), 2) including a quadratic age term to control for non-linear age effects (r = 0.998 with the original map), and 3) not winsorizing the input data (correlation of r = 0.987 with the original map). Next, we tested whether the observed prefrontal effect was present in different sub-samples. For this purpose, we drew 1000 sub-samples each containing 80% of the individuals and repeated the analysis 1000 times. The average correlation between the original t-map and the t-maps derived from the sub-samples was r = 0.929 (**Supplementary Figure S3B**).

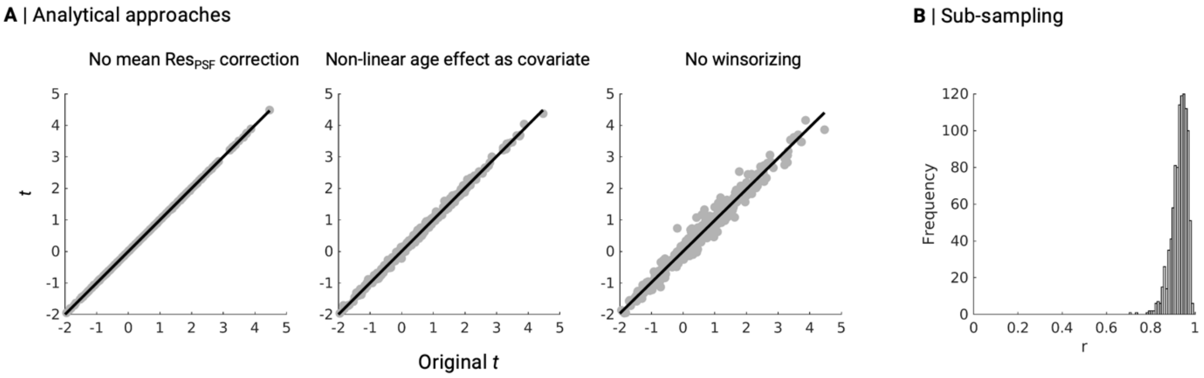

Supplementary Figure S3. ***Sensitivity tests for ΔRes$_{PSF}$ * ΔMT effects.*** **A)** Pearson's correlation between original the t-map (x-axis) presented in the main manuscript and t-maps derived from alternative analytical approaches (n = 141 individuals, all p < 0.001). **B)** Histogram depicting Pearson's correlations between the original t-map and t-maps computed based on 1000 randomly drawn sub-samples (80% of data).

Organizational axis of developmental change in MT

To assess whether ΔMT occurred in a synchronized manner across the cortex, we further observed that inter-regional covariance of ΔMT was organized along an anterior to posterior pattern (**Figure S4**). We then examined whether the topological distribution of Δ Res_{PSF} effects on ΔMT aligns with general organizational principles of intra-individual MT development and observed a positive correlation between the t-map and the previously identified principal axis of ΔMT ($r = 0.46$, $p_{spin} = 0.016$, CI = [0.36, 0.53]). Significance was assessed by a spin test (10000 permutations) correcting for spatial auto-correlations between cortical maps [2]. Thus, the association between change in resilient psychosocial functioning and MT maturation follows general organizational principles of MT development. This suggests that differences in the cortex-wide embedding of developmental change in anterior vs. posterior regions are reflected in the degree to which both apexes play a role in the development of resilient psychosocial functioning. The observed axis likely reflects differences in the timeline on which regions are most developmentally active with respect to myelination, and thus have differential relevance for adolescent resilience.

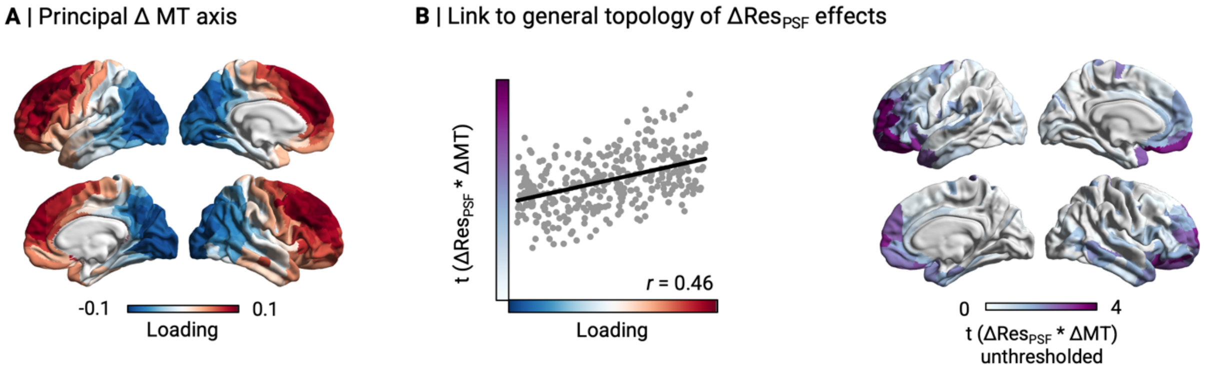

Supplementary Figure S4. ***Principal axis of synchronized MT change.*** **A)** Structural covariance of intra-individual deltas of Magnetic Transfer (ΔMT) was organized along an anterior-posterior cortical axis (n = 141 individuals). **B)** The unthresholded effect map reflecting the association between change in resilient psychosocial functioning (ΔRes_{PSF}) and ΔMT presented in the main manuscript was spatially correlated with the principal axis of MT development, as tested via a Pearson's correlation and significant after spin tests using 1000 rotations to control for spatial auto-correlation ($p_{spin} = 0.016$).

Cross-sectional effects of Res_{PSF} and Δ Res_{PSF}

We observed no cross-sectional association between baseline MT and baseline Res_{PSF}. However, we observed a negative effect of Δ Res_{PSF} on baseline MT in a parcel in the left middle frontal gyrus (L_p10p; t = -4.16, $p_{10,000 \text{ permutations + FDR}} < 0.05$). Thus, individuals who had lower Res_{PSF} at baseline compared to the follow-up time point also had cross-sectionally lower levels of MT in this region (**Figure S5**).

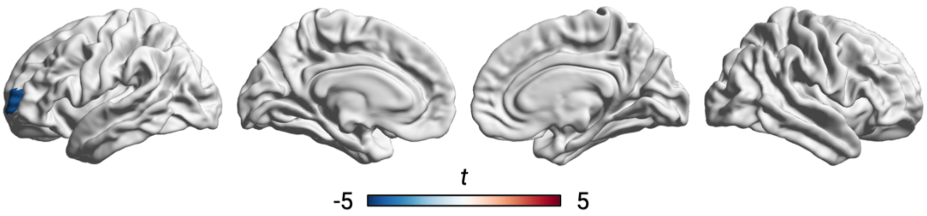

Supplementary Figure S5. ***Effects of change in resilient psychosocial functioning scores on baseline Magnetic Transfer (MT).*** P-values were corrected by 10.000 non-parametric permutations and FDR ($p < 0.05$; n = 141 individuals).

PFC sub-clusters in ΔFC analyses

Using functional connectivity data, we investigated whether regions that show differences in ΔMT as a function of ΔRes_{PSF} also exhibit different functional embedding. For this analysis, presented in the main manuscript, connectivity profiles were averaged across parcels that are part of the prefrontal ROI within each participant. As the mask/ROI includes large parts of the PFC and thus a nexus of different functional profiles, we further subdivided the cluster based on parcels' assignment to functional communities [3]. The ROI spanned sub-regions of frontoparietal, default mode, and limbic networks. Because large parts of the limbic network were excluded in FC analyses in this study due to low signal-to-noise ratios (see Methods), we tested associations between ΔRes_{PSF} and ΔFC only for frontoparietal and default mode sub-regions.

The prefrontal cluster showed predominantly negative change in FC across large parts of the frontal, temporal, and occipital cortex, as well as the insula and anterior cingulate cortex (**Supplementary Figure S6A**). Studying the association between ΔRes_{PSF} and ΔFC in sub-parts of the prefrontal cluster, it appeared that positive associations were largely driven by sub-regions that are part of the default mode network. That is, when defining sub-regions that are part of the frontoparietal network as seed, two regions (right medial PFC and PCC; **Supplementary Figure S6B**) showed significant effects after 10.000 permutations and FDR (alpha = 0.05). In contrast, defining sub-regions that are part of the default mode network as seed revealed positive associations between ΔRes_{PSF} and ΔFC in 21 regions, spanning medial and lateral PFC, posterior cingulate cortex, and parts of unimodal sensorimotor cortices (**Supplementary Figure S6C**).

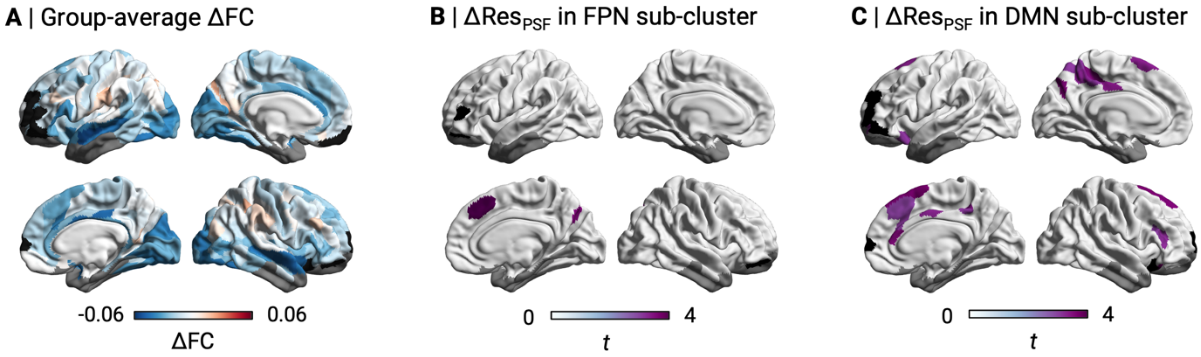

Supplementary Figure S6. ***Effects of change in resilient psychosocial functioning (ΔRes_{PSF}) scores on change in functional connectivity (FC) of sub-parts of the prefrontal region-of interest presented in the main analysis (n=141 individuals).*** A) Group-average pattern of change in FC (ΔFC) for the total prefrontal region of interest (ROI). B) We observed no significant association between ΔRes_{PSF} and ΔFC in the ROI sub-regions that are part of the frontoparietal network (FPN). C) Associations between ΔRes_{PSF} and ΔFC in ROI sub-regions that are part of the default mode network (DMN). B and C include age, sex, site, and mean Res_{PSF} as covariates. The respective ROIs are masked in black. Regions excluded due to low signal-to-noise ratio are masked in dark grey.

Robustness, estimated maturational processes, and future utility of the MPC maturational index

The maturational index based on microstructural profile similarity (MI_{MPC}) is a structural extension of the previously established functional connectivity maturational index, capturing conservative (i.e. strengthening of existing connections) and disruptive maturational modes (i.e., a reorganization of existing connectivity patterns [4]). While the functional maturational index identifies increasing levels of reorganization from unimodal (little reorganization) to transmodal cortex (more reorganization), the microstructural maturational index mirrors insights gained from a previously established cortical topology of synchronized age effects on microstructural profile covariance [5]. In the MI_{MPC}, we observe the strongest re-organization in frontoparietal heteromodal cortex, whereas a "frame" of ventral/paralimbic and dorsal/somatosensory cortex shows mostly an age-related strengthening of existing MPC patterns (i.e., little re-organization). This is consistent with what Paquola & Bethlehem et al. reported: association cortical areas, in which overall intra-cortical myelin content increases, develop towards a more "sensory" architecture, whereas regions in which preferably mid-to-deeper layers show increases in myelin develop towards a more "paralimbic" architecture. This differentiation process is reflected in the 'disruptive re-organization' captured by the MI_{MPC}, and mirrors the modular segregation observed in tractography-based adolescent data [6]. At the same time, paralimbic-temporal/ventral and somatosensory/dorsal regions show 'conservative development' (i.e., little re-organization) in the MI_{MPC}, suggesting that MPC patterns are well-defined prior to adolescence [5,7]. Overall, the MI_{MPC} pattern meaningfully captures synchronized microstructural maturation and re-organization, consistent with previous observations.

To probe the robustness of the MI derived from microstructural data, we repeated the computation of the MI_{MPC} based on different subsamples. First, we drew 100 sub-samples, each containing 80% of the individuals per NSPN age bin and repeated the analysis 100 times. The average correlation between the MI_{MPC} map based on all individuals for whom MT data was available (n = 295 subjects, 512 sessions/datapoints) and MI_{MPC} maps derived from 80%-sub-samples was r = 0.96 (**Supplementary Figure S7A**). Next, we assessed whether the MI_{MPC} map can be observed with smaller sample sizes. To this end, we again drew sub-samples per age bin, but this time the size of the subsamples ranged between 20% and 100% (in steps of 5%) of individuals (see **Supplementary Figure S7B**). Last, results stayed consistent when computing the MI_{MPC} – which reflects the correlation between baseline and age-related change patterns – based on Spearman's or Pearson's correlation ($r = 0.99$; $p_{spin} < .0001$) **Supplementary Figure S7C**).

The maturational index based on microstructural profile covariance adds to our understanding of inter-regionally synchronized cortical maturation, capturing adolescent re-organization (integration and segregation) of primarily frontoparietal association cortex. That is, emerging work highlights integrated multi-scale approaches to elucidate biological risk factors associated with neuropsychiatric conditions. It is increasingly recognized that pathological functional perturbations are coupled with microstructural perturbations [8–11]. Moreover, taking a nuanced approach to studying intracortical myeloarchitectural profiles, beyond mean myelin content, has revealed parallel maturational processes at different scales and topologies [5,12,13]. The use of the MI_{MPC} in future studies may mirror the current use of similar, already established measures such as the main axis of MPC age effects and the maturational index for functional connectivity. That is, the main axis of MPC age effects has already been linked to the cortical topology from microstructural profiles and histology [5], which in tun has been combined with other measures of 'cortical wiring' in adolescence [14]. Assessing the topology of synchronized structural maturation based on intra-cortical profiles further extends classical structural covariance approaches, assessing e.g. cortical thickness covariance [15], towards a more nuanced myeloarchitecture. Last, similar to our study, the previously established maturational index of functional

connectivity [4] has been demonstrated to robustly capture sex differences in adolescent functional network maturation [16]. In summary, the MI_{MPC} may be of interest for future studies of adolescent cortical maturation.

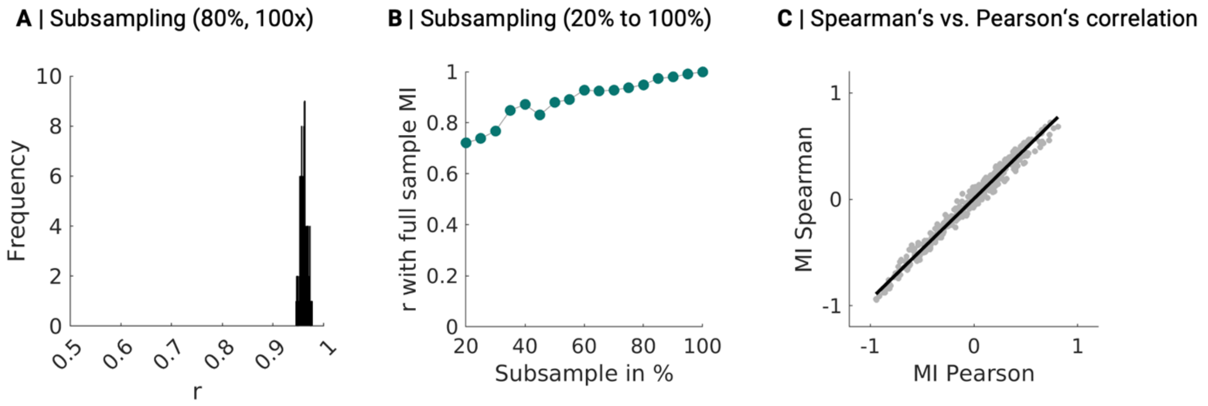

Supplementary Figure S7. ***Robustness of the MPC maturational index.*** **A)** Histogram depicting correlations between the microstructural profile covariance maturational index (MI_{MPC}) derived from all n = 295 individuals (512 sessions) and the MI_{MPC} based on 100 sub-samples (80% of data). **B)** Correlations between the MI_{MPC} derived from all 295 individuals (512 sessions) and the MI_{MPC} based on subsamples of different sizes, ranging from 25-100% in steps of 5%. **C)** The MI is computed as the correlation between baseline and change patterns. Here, we applied both Spearman's and Pearson's correlation to compute the MI and correlated the MI pattern resulting from either method via a Pearson's correlation (r = 0.99, p_{spin} <.0001).

Conservative vs. disruptive trends MI_{MPC} Group differences

We tested for differences in the maturational index (MI) between groups of individuals who showed increasingly resilient vs. susceptible outcomes. **Table S3** summarizes all 43 parcels displaying significant group differences in MI_{MPC} (as determined both via z-tests and non-parametric permutation testing). Trends were determined with respect to the full sample MI_{MPC} presented in Figure 2. For example, a positive ΔMI_{MPC} in a region labeled 'conservative' in the full sample MI_{MPC} is interpreted as '+ΔRes_{PSF} more conservative', whereas a positive ΔMI_{MPC} in a region labeled 'disruptive' in the full sample is interpreted as '+Res_{PSF} less disruptive'. Regions that show a significant group difference but neither a clear conservative nor disruptive pattern in the full sample are labeled as 'tipping points'.

Table S3. ROIs with significant group differences in MI_{MPC} and their trends. Group differences were derived from z-tests and adjusted for multiple comparisons by thresholding at pFDR <0.05 as well as non-parametric permutation testing using 10.000 permutations. Tests were two-sided.

ROI	ΔMI_{MPC}	z	Trend
L_RSC	-0.507	-7.09	tipping points
L_31pv	-0.565	-9.51	+ΔRes_{PSF} more disruptive
L_7AL	-0.28	-3.79	+ΔRes_{PSF} less conservative
L_7PL	-0.619	-8.63	tipping points
L_9p	-0.37	-5	tipping points
L_10d	-0.564	-8.41	+ΔRes_{PSF} more disruptive
L_IFSa	-0.579	-13	+ΔRes_{PSF} more disruptive
L_PoI2	-0.262	-3.52	+ΔRes_{PSF} less conservative
L_TE2a	-0.352	-4.82	+ΔRes_{PSF} less conservative
L_TF	-0.388	-5.49	+ΔRes_{PSF} less conservative
L_IP1	-0.694	-9.92	+ΔRes_{PSF} less conservative
L_PGi	-0.938	-15.1	+ΔRes_{PSF} more disruptive
L_V6A	-1.044	-16.8	+ΔRes_{PSF} less conservative
L_s32	-0.617	-8.53	+ΔRes_{PSF} less conservative
L_Ig	-0.54	-12.1	+ΔRes_{PSF} more disruptive
L_p10p	0.525	9.98	+ΔRes_{PSF} less disruptive
R_RSC	-0.468	-6.38	+ΔRes_{PSF} less conservative
R_FFC	-0.553	-8.01	+ΔRes_{PSF} less conservative
R_SFL	-0.225	-3.02	+ΔRes_{PSF} less conservative
R_PCV	-0.426	-7.07	+ΔRes_{PSF} more disruptive
R_7Pm	-0.694	-13.1	tipping points
R_v23ab	-1.021	-15.1	tipping points
R_24dv	1.003	17.3	+ΔRes_{PSF} less disruptive
R_7Am	-0.802	-11.3	+ΔRes_{PSF} less conservative
R_7PL	-0.713	-10.2	+ΔRes_{PSF} less conservative
R_p32	-0.543	-7.52	+ΔRes_{PSF} less conservative
R_10r	-0.935	-13.6	+ΔRes_{PSF} less conservative

R_47m	-0.549	-8.42	+ΔRes$_{PSF}$ more disruptive
R_OFC	-0.618	-8.67	+ΔRes$_{PSF}$ less conservative
R_6a	0.625	16.1	+ΔRes$_{PSF}$ less disruptive
R_PFcm	-0.846	-13.7	tipping points
R_FOP4	-0.707	-9.88	+ΔRes$_{PSF}$ less conservative
R_A5	-0.621	-9.15	+ΔRes$_{PSF}$ less conservative
R_STSda	-0.839	-12	+ΔRes$_{PSF}$ less conservative
R_TE1a	-0.667	-9.27	+ΔRes$_{PSF}$ less conservative
R_TE2a	-0.552	-7.72	+ΔRes$_{PSF}$ less conservative
R_TPOJ1	-0.76	-11	+ΔRes$_{PSF}$ more disruptive
R_V6A	-1.098	-18.5	tipping points
R_VMV1	-0.97	-15.7	+ΔRes$_{PSF}$ less conservative
R_pOFC	-0.186	-2.65	+ΔRes$_{PSF}$ less conservative
R_FOP5	-0.483	-6.59	+ΔRes$_{PSF}$ less conservative
R_LBelt	-0.312	-4.35	+ΔRes$_{PSF}$ less conservative
R_TE1m	-0.404	-5.5	+ΔRes$_{PSF}$ less conservative

+Δ Res$_{PSF}$ = Increasingly resilient psychosocial functioning with age. ROI labels refer to the HCP parcellation [17]

Testing group differences in MI_{MPC} via alternative modeling approaches.

To test the robustness of the observed group differences in MI_{MPC} to an alternative modeling approach, we also combined the groups in a joint interaction model (see Supplementary Methods). We observed a high correlation between group-difference maps derived from the original, group-stratified analysis compared to the alternative analysis based on a fixed term for the group-by-age interaction (r = 0.97; **Supplementary Figure S8A**). Thus, we concluded that the results were independent from the modeling approach, however, we consider the group-stratified approach more intuitive. Furthermore, the results remained consistent when computing the MI_{MPC} – which reflects the correlation between baseline and age-related change patterns – based on Spearman's or Pearson's correlation (r = 0.99; **Supplementary Figure S8B**). Next, we tested whether the observed topology of group differences in MI_{MPC} stay consistent in sub-samples. To this end, we drew 1000 sub-samples each containing 80% of the individuals and repeated the analysis 1000 times. The average correlation between the group difference map based on 141 individuals and the group difference maps derived from sub-samples was r = 0.85 (**Supplementary Figure S8C**).

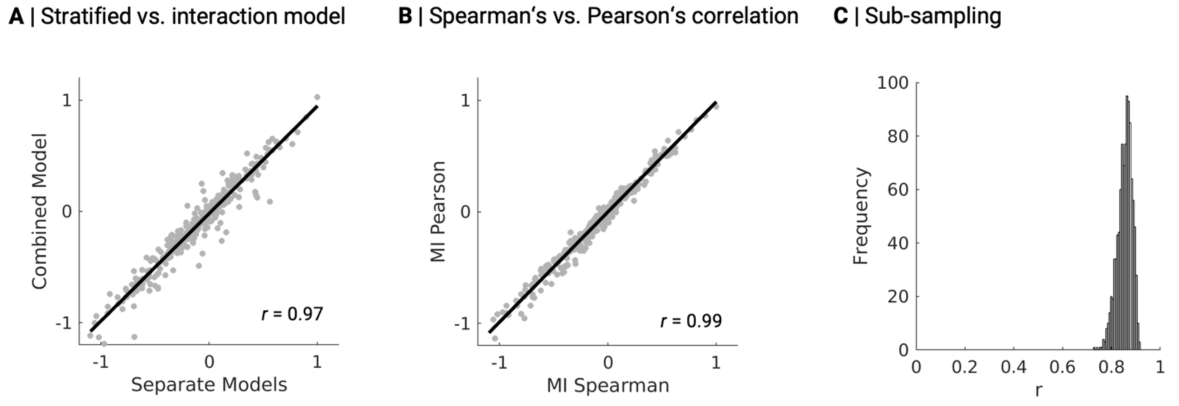

Supplementary Figure S8. ***Group differences in the maturational index (MI_{MPC}) based on alternative modeling approaches.*** **A)** Pearson's correlation between group differences in MI_{MPC} modeled in separate models compared to a joint interaction model (n = 141 individuals; $p_{spin} < .0001$). **B)** The MI is computed as the correlation between baseline and change patterns. Here, we applied both Spearman's and Pearson's correlation to compute the MI per group and correlated the group difference pattern resulting from either method using a Pearson's correlation ($p_{spin} < .0001$) **C)** Histogram depicting Pearson's correlations between the group difference map based on all 141 individuals and group difference maps computed based on 1000 randomly drawn sub-samples (80% of data).

FC Maturational index in increasingly resilient vs. increasingly susceptible psychosocial functioning

We observed a decoupling of microstructural and functional connectivity MIs in large parts of the heteromodal cortex (see Figure 3G). For completeness, we therefore also tested for group differences in MI_{FC} between individuals who developed towards more resilient vs. more susceptible outcomes as well, following the same analytical steps as described for MI_{MPC}. We observed significant but more subtle group differences in 8 limited, primarily prefrontal regions (**Supplementary Figure S9A**). The results were robust to alternative modeling approaches (**Supplementary Figure S9B-D**).

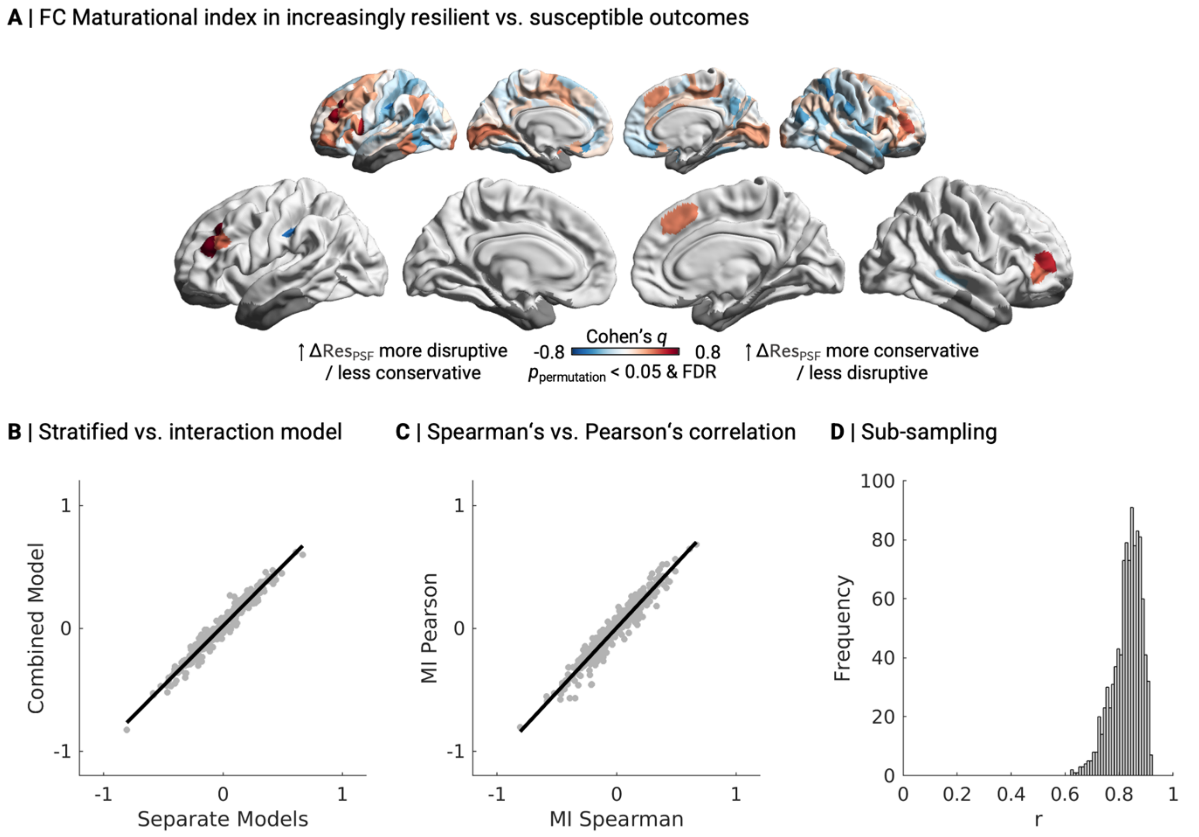

Supplementary Figure S9. ***Regionally less disruptive development of functional connectivity networks with increasingly resilient psychosocial functioning.*** **A)** Group differences in the functional connectivity Maturational Index (MI_{FC}; n = 141 individuals). In this visualization, the MI_{FC} of the group of individuals becoming more susceptible with age was subtracted from the MI_{FC} of the group of individuals becoming more resilient with age (↑ ΔRes_{PSF}), and thresholded at $p<0.05$ (FDR & 10,000 permutations). The smaller brain plot on top depicts the unthresholded group difference map. Parcels masked in dark grey were excluded due to low signal-to-noise ratios. **B-D)** depict sensitivity checks using alternative modeling approaches and sub-sampling. **B)** Pearson's correlation between group differences in MI_{FC} modeled in separate models (stratified approach) compared to a joint interaction model ($r = 0.98$, $p<0.0001$. **C)** The MI is computed as the correlation between baseline and change patterns. Here, we applied both Spearman's and Pearson's correlation to compute the MI_{FC} per group and correlated the resulting group difference patterns ($r = 0.96$, $p<0.0001$). **D)** Histogram depicting correlations between the group difference map based on all 141 individuals and group difference maps computed based on 1000 randomly drawn sub-samples (80% of data; average Pearson's correlation: 0.83).

Supplementary Discussion

Insights gained and potential sources of noise related to using residuals as resilience scores

Assessing resilient/susceptible outcomes based on the deviation of observed from expected levels of well-being given a certain adversity exposure is a well-established approach in the resilience literature [18–23]. It addresses the issue that simple quantification of mental health variables can provide only limited insights into resilience or susceptibility, as they are heavily conflated with individual differences in adversity exposure (Kalisch et al., 2021). Leveraging residuals to quantify better or worse well-being than predicted by adversity exposure thus provides a corrected well-being score that is adjusted for individual differences in stressor exposure and thus allows comparing resilience scores of individuals with differing exposure levels. That is, 'residuals' in the resilience use case are interpreted as 'residual variance in mental health problems' that is not explained by the normative response to exposure, and therefore indicate individually weaker response (resilience) or a stronger response (susceptibility). It is thus analogous to the process of correcting a dependent variable for potential confounders such as age or sex.

While the basic assumption of this approach is that residualized mental health outcomes reflect degrees of resilience/susceptibility, there are other potential influences such as 1) measurement error and noise related to the questionnaires used, 2) noise related to the performance of our prediction model, and 3) confounding influences of other environmental influences.

1) Self-report / retrospectivity biases may pose one source of measurement error, e.g. for the reporting of childhood maltreatment [25], despite the relatively short reporting time windows of the current study. It should be noted, however, that such sources of measurement error are not unique to / caused by the residual approach, but rather persist from the original measures of psychosocial well-being. The resilience scores should therefore not contain more measurement error than the original measures of psychosocial well-being. In the current study, our longitudinal approach may further mitigate some aspects of measurement error if it is linked to e.g., self-report bias. That is, an individual systematically self-reporting his/her well-being as a bit better than it is will receive higher resilience scores at all time points. However, as our study investigates intra-individual change, such a general offset in resilience scores would not affect change scores, whereas it would affect cross-sectional analyses more strongly.
2) Another question is how well our prediction model controls for differences in adversity exposure in order to reveal resilient / susceptible responses – and to what extend influence is still uncontrolled for, thus causing noise in in our measure of individual resilience. The amount of variance explained by our model (approx. 21%) is comparable with previous work reporting 24% of variance explained for the prediction of psychosocial functioning from family experiences in longitudinal settings [23]; 21% when predicting internalizing symptoms from general life stressors during the covid pandemic [26]; or 28% when predicting psychosocial functioning from childhood adversity [27]. We thus conclude that our model controls for exposure to a comparable degree as common resilience models.
3) Capturing meaningful variation in psychological outcomes is complicated by the complex influence of a multitude of interacting factors, which likely contribute to the variance not explained by our model. Such factors may include genetic predispositions, other environmental risk or protective factors not measured here, but likely also sources of noise we cannot quantify. For instance, an individual with a genetic predisposition for mental illness may systematically show lower psychological well-being, which cannot fully be explained by adversity exposure and would thus create a bias in that individuals' derived resilience scores. Similar to 1), if general offsets in

resilience scores exist due to e.g., a genetic predisposition, studying longitudinal change helps us to account for such an offset.

Overall, our results using resilient psychosocial functioning scores suggest a central role for multi-modal prefrontal maturation and more wide-spread re-organization of association cortices for resilience and susceptibility during adolescence, tested against null models by non-parametric permutation. This observation is well in line with previous reports of structural and functional involvement of these brain regions in stress responses and susceptibility/resilience [28–32]. We believe the fact that observed associations of residuals / resilience scores with multi-modal measures of cortical maturation survived non-parametric permutation tests, and were found in regions previously suggested by the literature, argues for a dominance of meaningful variance reflected in the scores used.

Supplementary Methods

Sample

The NeuroScience in Psychiatry (NSPN; [33]) Cohort was recruited via NHS primary care services, schools, colleges, and direct advertisement for five sex and ethnicity-balanced age bins (14-15, 16-17, 18-19, 20-21, and >22). This 'NSPN 2K Cohort' completed demographic and medical, as well as mental health related assessments via home questionnaire packs. A 'U-change' MRI cohort (n = 318) subsample completed structural and functional scanning in either London or Cambridge, UK. All participants aged 16 years and over gave informed consent. Participants younger than 16 years gave informed assent, and consent was provided by their parent or guardian. Demographics of different sub-samples used in this study are presented in **Supplementary Figure S10**. Although neuroimaging was only conducted in a sub-set of individuals, we performed behavioral analyses on the entire NSPN sample to maximize training data for Res_{PSF} computation.

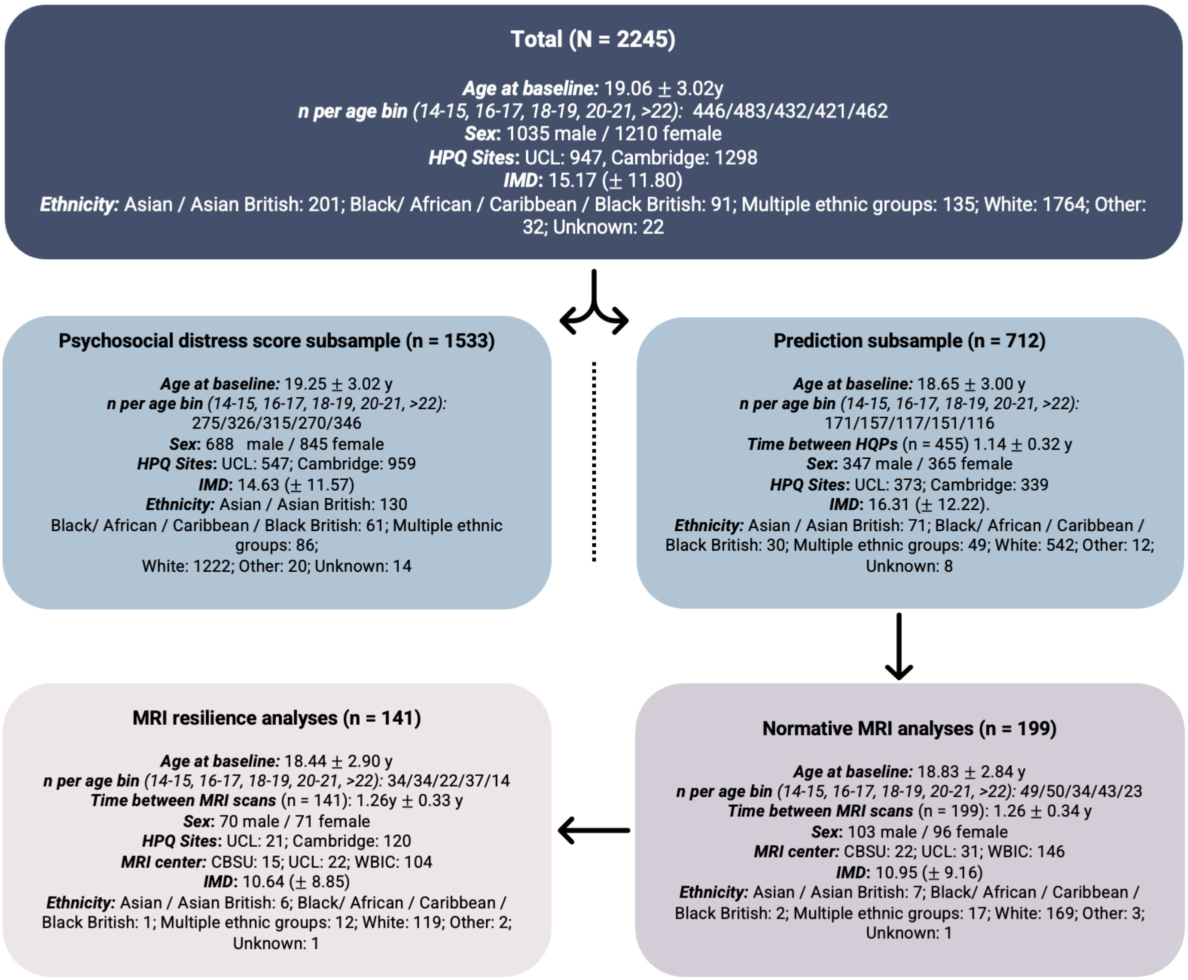

Figure S10. ***Demographic information for different subsamples included in presented analyses.*** HPQ = Home Questionnaire Pack; UCL = University College London; IMD = Indices of multiple deprivation; WBIC: Wolfson Brain Imaging Centre, Cambridge; CBSU: MRC Cognition and Brain Sciences Unit, Cambridge; UCL: University College London, London

*Sensitivity tests for ΔRes_{PSF} * ΔMT association*

We tested whether the observed association between ΔRes_{PSF} and ΔMT was robust to analytical choices. Alternative models assessed were:

1. not including mean Res_{PSF} as a covariate in the general model (1):

(1)

$$\Delta MT(parcel) \sim 1 + \beta_{\Delta Res\_PSF} * \Delta Res_{PSF} + \beta_{age} * age + \beta_{sex} * sex + \beta_{site} * site,$$

2. Including a quadratic age term to control for non-linear effects (2):

(2)

$$\Delta MT(parcel) \sim 1 + \beta_{\Delta Res\_PSF} * \Delta\ Res_{PSF} + \beta_{mean\ Res\_PSF} * mean\ Res_{PSF} + \beta_{age} * age + \beta_{age^2} * age^2 + \beta_{sex} * sex + \beta_{site} * site,$$

3. Not winsorizing the input data.

Main axis of ΔMT development

For a system-level understanding of how MT change is synchronized across the cortex, we derived a principal axis of microstructural group-level covariance. To this end, we first computed pairwise correlations between ΔMT values between all pairs of regions, across individuals. This served as an indicator of the degree to which the slopes of ΔMT are similar between any two regions. Next, we applied diffusion map embedding, a nonlinear dimensionality reduction technique [34], to the derived matrix, to capture the spatial topography of synchronized MT change on a unidimensional axis. Regions at the peaks of the derived axis reflect maximally different embeddings of MT change, whereas regions closer together on this axis change in a similar manner, across individuals.

In order to assess whether associations between ΔRes_{PSF} and ΔMT follow the overall organizational axis of ΔMT, we correlated the unthresholded t-map with the principal axis. Significance of this correlation was assessed via spin-tests which correct for spatial auto-correlations (10.000 spins; [2]).

Psychosocial distress score questionnaire information

Following scoring procedures described in St Clair et al. [35], we used the following response system: RSE, ABQ, r-LOI, and RCMAS [36–38] were scored in a four-level response system ('never', 'sometimes', 'mostly', and 'always') in which individuals indicated how frequently the emotion or behavior described by an item applied to them within the previous two weeks. All but five RSE items were negatively worded, but they were not reversed for the factor analysis. For the ABQ, the large majority of individuals did not ever select the 'mostly' and 'always' categories, which is why responses were binarized to 'never' and 'sometimes/mostly/always'.

The Warwick-Edinburgh Mental Wellbeing Scale (WEMWBS; [39]) is a 14-item questionnaire that measures mental well-being in a positively worded fashion. Participants were asked to respond on a 5-point Likert scale ('none of the time', 'rarely', 'some of the time', 'often', 'all of the time') to which degree statements described their experiences in the last two weeks. Example items are 'I've been feeling good about myself', 'I've been feeling useful', I've been feeling close to other people'. A sum score was used for present analyses.

The Schizotypal Personality Questionnaire (SPQ; [40]) originally includes 74 binary ('present' or 'absent') self-report items designed to capture symptoms associated with the DSM-III definition of Schizotypal Personality disorder, such as psychotic-like experiences. We included only items that have previously been tested to be significantly associated and showing medium to high effect sizes with psychotic-like experiences as measured by the semi-structured PLIKS interview (PLIKSi; total score, hallucinations, delusions, and perceptual abnormalities; [35,41]. Items retained based on this face validity include: SPQ 4, 9, 13, 28,31, 40, 55, 60, 61, 63 and 64.

Adversity questionnaires information

The Life Events Questionnaire (LEQ; [42] asks individuals about significant life events that have occurred within the previous 18 months. Such significant life events include changing schools / college / jobs, moving, changes in family composition like death or divorce, disasters at home (e.g. fire), serious illness and/or hospitalization of self or someone in the close network of family and friends, deaths, loss of family pet, problems with or end of friendships, and others. Participants were instructed to focus on the most impactful event if they experienced multiple situations captured by the same category. Moreover, participants rated how (un)pleasant the event was ('very pleasant', 'pleasant', 'neither', 'quite unpleasant', 'very unpleasant') and whether it impacted them for more than 2 weeks. For our analyses, we used the LEQ sum score capturing how many adverse life events (i.e., events scored as 'quite unpleasant' or 'very unpleasant') an individual faced within the given time period.

The Child Trauma Questionnaire (CTQ; [43]) measures abuse and neglect up to age 18 years on a five-point Likert scale ('never' to 'always') in five overarching categories: emotional abuse (e.g., 'family members called me stupid, lazy, or ugly'), physical abuse (e.g., 'someone from my family hit me so hard that I had to see a doctor or go to the hospital'), sexual abuse (e.g., 'someone tried to touch me in a sexual way or made me touch him/her in a sexual way'), emotional neglect ('e.g., I felt loved'), physical neglect (e.g., 'someone took me to the doctor when it was necessary'). Each category contains five items. Items 2,5,7,13,19,26, and 28 were reversed before taking the sum score for current analyses. If data was missing for one time point but was available for other timepoints, the missing datapoint was imputed for that subject based on the average of the remaining time points. Imputation was done within the five categories separately (sessions / % per category: emotional abuse: 15/0.0054, physical abuse: 4/0.0014, sexual abuse: 4/0.0014, emotional neglect: 17/0.0061, physical neglect: 19/0.0068). Moreover, the CTQ was part of the questionnaires given only to participants of the U-change cohort, meaning that the measurement time point did not match that of other questionnaires. As CTQ items are also less timepoint specific compared to e.g., the LEQ, we used the average CTQ rating across sessions for our analyses.

The Alabama Parenting Questionnaire (APQ) as included in the NSPN study contains 15 items asking about parenting styles. These 15 items are a combination of 9 items from the original version of the APQ [44], the Corporal Punishment scale (3 items), and the Involvement scale (3 items). Participants rated the frequency of occurrences of certain parenting styles in their family on a five-point scale

(‘never’ to ‘always), asking about positive parenting (3 items), inconsistent discipline (3), poor supervision (3 items), involvement (3 items), and corporal punishment (3 items). We reversed the ‘positive parenting’ and ‘involvement’ items so that higher scores reflect more adverse parenting styles. Imputation was performed in the same way as described for the CTQ. Imputed sessions/% per category: positive parenting: 30/0.0054, inconsistent parenting: 51/0.0062, poor supervision: 48/0.0086, involvement: 50/0.0090, corporal punishment: 12/0.0022.

The Measure of Parenting Style (MOPS; [45]) measures dysfunctional parenting practices in 30 items, 15 for each parent. The questionnaire covers indifference/neglect (6 items, e.g., ’Was uninterested in me’), over-control (4 items, e.g., ‘Sought to make me feel guilty’), and abuse (5 items, e.g. ’Physically violent or abusive of me ‘). Higher scores reflect more dysfunctional parenting styles in all sub-scales. Scores were first summed within categories and then across categories for the current analyses.

Socioeconomic status was indirectly derived from the Index of Multiple Deprivation (IDM) associated with the area a participant lived in. The MDI is based on regional income, employment rate, education, health and health service, crime rates, barriers to housing and serviced, living environment and others. IDMs were imputed based on the mean if missing (15 sessions = 0.01%).

Cross-sectional effects of Res_{PSF} and Δ Res_{PSF} on MT

In order to assess whether the observed positive association between ΔRes_{PSF} and ΔMT in the prefrontal cortex co-occurs with 1) pre-existing hypo- or hypermyelination, or 2) cross-sectional differences in baseline MT as a correlate of Res_{PSF}, we applied two general linear models to baseline MT data:

(3)

$$MT_{baseline}(parcel) \sim 1 + \beta_{\Delta\ Res\_PSF} * \Delta\ Res_{PSF} + \beta_{Res\_PSF\_mean} * Res_{PSF}\_mean + \beta_{age} * age + \beta_{sex} * sex + \beta_{site} * site,$$

and

(4)

$$MT_{baseline}(parcel) \sim 1 + \beta_{Res\_PSF\_baseline} * Res_{PSF\_\ baseline} + \beta_{age} * age + \beta_{sex} * sex + \beta_{site} * site,$$

Non-parametric permutations for +/- Δ Res_{PSF} group allocations

Comparing maturational index (MI) patterns between individuals with increasingly resilient vs. vulnerable outcomes, the sample had to be divided into two groups. We followed previously published procedures comparing regional MI patterns between two groups via z-tests [16]. To further control for sampling bias, we tested differences in MI against 10,000 null-models derived by shuffling participant group allocations 10,000 times. In each permutation, repeated sessions of the same individual were allocated together, group size imbalances and distributions were controlled to resemble the original groups, and permutations were performed within age groups to maintain the stratified design of the NSPN cohort upright (see **Supplementary Figure S10)**.

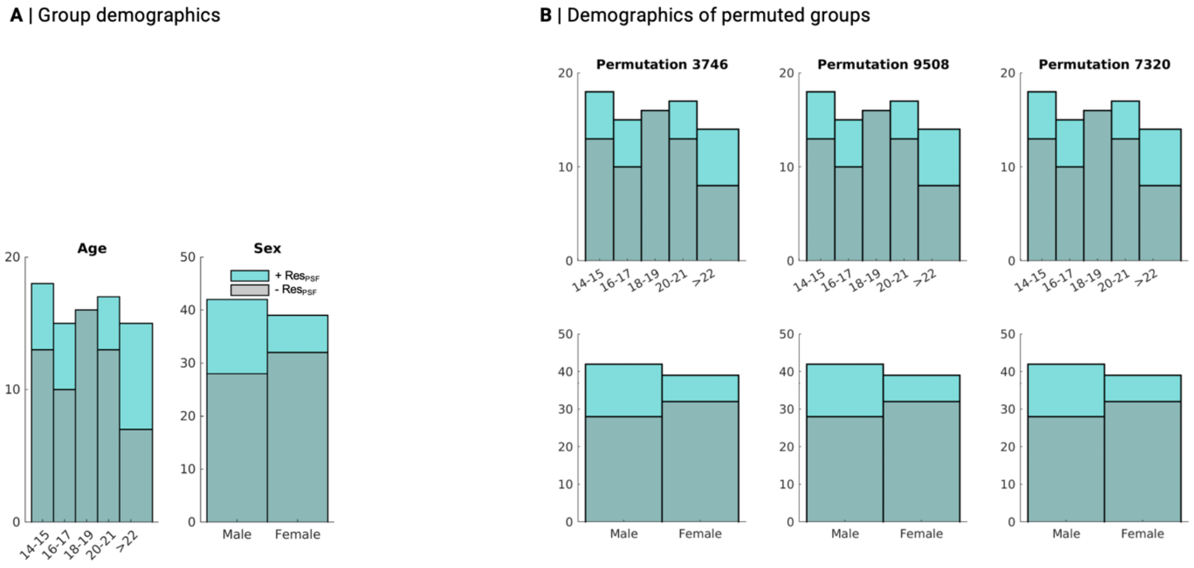

Supplementary Figure S11. ***Age and sex distributions per group in the original (A) and example permuted (B) data (n=141 individuals).***

Maturational index group differences via interaction model

To test the robustness of the observed group differences in the MI_{MPC} between +Δ vs. -Δ Res_{PSF} groups to an alternative modeling strategy, we also analyzed the main effects of age and Δ Res_{PSF} group, and the age-by-ΔRes_{PSF} group interaction, in the full data (i.e., both groups combined) using a linear mixed effects model at each edge:

(5)

$$MPC(k,j) \sim 1 + \beta_{\Delta Res\_PSF\text{-}group} * \Delta\, Res_{PSF}\text{-group} + \beta_{age} * age + \beta_{age*\Delta Res\_PSF\text{-}group} * age * \Delta Res\_PSF\text{-group} + \beta_{sex} * sex + \beta_{site} * site + \gamma_{subject} * (1|subject) + \epsilon$$

where MPC(k,j) refers to the MPC at edge level, β refers to coefficients for the fixed effects, $\gamma_{subject}$ refers to coefficients for random effects and ϵ represents the residual error.

We then estimated MPC_{14} for +ΔRes_{PSF} and -ΔRes_{PSF} groups as follows:

(6)

$$MPC14_{+\Delta Res\_PSF} = 1 + \beta_{\Delta Res\_PSF\text{-}group} *1 + \beta_{age} * 14 + \beta_{age*\Delta Res\_PSF\text{-}group}*14*1 + \beta_{sex} * (1/2) + \beta_{site1} *(1/3) + \beta_{site2} *(1/3)$$

(7)

$$MPC\ 14_{-\Delta Res\_PSF} = 1 + \beta_{\Delta Res\_PSF\text{-}group} *0 + \beta_{age} * 14 + \beta_{age*\Delta Res\_PSF\text{-}group}*14*0 + \beta_{sex} * (1/2) + \beta_{site1} *(1/3) + \beta_{site2} *(1/3)$$

And then estimated $MPC_{14\text{-}26}$ for the +Δ Res_{PSF} and -Δ Res_{PSF} groups:

(8)

$$MPC_{14\text{-}26\ +\Delta Res\_PSF\text{-}group} = \beta age + \beta_{age*\Delta Res\_PSF\text{-}group} * 1$$

(9)

$$MPC_{14\text{-}26\text{-}\Delta Res\_PSF\text{-}group} = \beta age + \beta_{age*\Delta Res\_PSF\text{-}group} * 0$$

Finally, like in the main analysis, we computed row-wise Spearman's correlations between extracted MPC_{14} and $MPC_{14\text{-}26}$ data for each group separately.

NSPN Consortium Member List

Principal investigators:
Edward Bullmore (CI from 01/01/2017)
Raymond Dolan
Ian Goodyer (CI until 01/01/2017)
Peter Fonagy
Peter Jones

NSPN (funded) staff:
Michael Moutoussis
Tobias Hauser
Sharon Neufeld
Rafael Romero-García
Michelle St Clair
Petra Vértes
Kirstie Whitaker
Becky Inkster
Gita Prabhu
Cinly Ooi
Umar Toseeb
Barry Widmer
Junaid Bhatti
Laura Villis
Ayesha Alrumaithi
Sarah Birt
Aislinn Bowler
Kalia Cleridou
Hina Dadabhoy
Emma Davies
Ashlyn Firkins
Sian Granville
Elizabeth Harding
Alexandra Hopkins
Daniel Isaacs
Janchai King
Danae Kokorikou
Christina Maurice
Cleo McIntosh
Jessica Memarzia
Harriet Mills
Ciara O'Donnell
Sara Pantaleone
Jenny Scott

Affiliated scientists:
Pasco Fearon
John Suckling
Anne-Laura van Harmelen
Rogier Kievit
Richard Bethlehem

Supplementary References

1. Garcini, L. M. *et al.* Increasing diversity in developmental cognitive neuroscience: A roadmap for increasing representation in pediatric neuroimaging research. *Dev. Cogn. Neurosci.* **58**, 101167 (2022).
2. Alexander-Bloch, A. F. *et al.* On testing for spatial correspondence between maps of human brain structure and function. *Neuroimage* **178**, 540–551 (2018).
3. Yeo, B. T. *et al.* The organization of the human cerebral cortex estimated by intrinsic functional connectivity. *J. Neurophysiol.* (2011).
4. Váša, F. *et al.* Conservative and disruptive modes of adolescent change in human brain functional connectivity. *Proc. Natl. Acad. Sci.* **117**, 3248–3253 (2020).
5. Paquola, C. *et al.* Shifts in myeloarchitecture characterise adolescent development of cortical gradients. *eLife* **8**, (2019).
6. Baum, G. L. *et al.* Modular Segregation of Structural Brain Networks Supports the Development of Executive Function in Youth. *Curr. Biol.* **27**, 1561-1572.e8 (2017).
7. Grydeland, H. *et al.* Waves of Maturation and Senescence in Micro-structural MRI Markers of Human Cortical Myelination over the Lifespan. *Cereb. Cortex N. Y. N 1991* **29**, 1369–1381 (2019).
8. Lariviere, S. *et al.* Microstructure-Informed Connectomics: Enriching Large-Scale Descriptions of Healthy and Diseased Brains. *Brain Connect* **9**, 113–127 (2019).
9. Park, B.-Y. *et al.* Differences in subcortico-cortical interactions identified from connectome and microcircuit models in autism. *Nat. Commun.* **12**, (2021).
10. Yang, G. J. *et al.* Functional hierarchy underlies preferential connectivity disturbances in schizophrenia. *Proc. Natl. Acad. Sci.* **113**, E219–E228 (2016).
11. Zheng, Y.-Q. *et al.* Local vulnerability and global connectivity jointly shape neurodegenerative disease propagation. *PLOS Biol.* **17**, e3000495 (2019).
12. Whitaker, K. J. *et al.* Adolescence is associated with genomically patterned consolidation of the hubs of the human brain connectome. *Proc. Natl. Acad. Sci.* **113**, 9105–9110 (2016).
13. Ziegler, G. *et al.* Compulsivity and impulsivity traits linked to attenuated developmental frontostriatal myelination trajectories. *Nat. Neurosci.* **22**, 992–999 (2019).
14. Park, B. *et al.* Adolescent development of multiscale structural wiring and functional interactions in the human connectome. *Proc. Natl. Acad. Sci.* **119**, e2116673119 (2022).
15. Raznahan, A. *et al.* Patterns of Coordinated Anatomical Change in Human Cortical Development: A Longitudinal Neuroimaging Study of Maturational Coupling. *Neuron* **72**, 873–884 (2011).
16. Dorfschmidt, L. *et al.* Sexually divergent development of depression-related brain networks during healthy human adolescence. *Sci. Adv.* **8**, eabm7825 (2022).
17. Glasser, M. F. *et al.* A multi-modal parcellation of human cerebral cortex. *Nature* **536**, 171–178 (2016).
18. Bowes, L., Maughan, B., Caspi, A., Moffitt, T. E. & Arseneault, L. Families promote emotional and behavioural resilience to bullying: evidence of an environmental effect. *J. Child Psychol. Psychiatry* **51**, 809–817 (2010).
19. Collishaw, S. *et al.* Mental health resilience in the adolescent offspring of parents with depression: a prospective longitudinal study. *Lancet Psychiatry* **3**, 49–57 (2016).
20. Kalisch, R. *et al.* The resilience framework as a strategy to combat stress-related disorders. *Nat. Hum. Behav.* **1**, 784–790 (2017).
21. Miller-Lewis, L. R., Searle, A. K., Sawyer, M. G., Baghurst, P. A. & Hedley, D. Resource factors for mental health resilience in early childhood: An analysis with multiple methodologies. *Child Adolesc. Psychiatry Ment. Health* **7**, 6 (2013).
22. Sapouna, M. & Wolke, D. Resilience to bullying victimization: the role of individual, family

and peer characteristics. *Child Abuse Negl.* **37**, 997–1006 (2013).
23. Van Harmelen, A.-L. *et al.* Adolescent friendships predict later resilient functioning across psychosocial domains in a healthy community cohort. *Psychol. Med.* **47**, 2312–2322 (2017).
24. Kalisch, R. *et al.* The Frequent Stressor and Mental Health Monitoring-Paradigm: A Proposal for the Operationalization and Measurement of Resilience and the Identification of Resilience Processes in Longitudinal Observational Studies. *Front. Psychol.* **12**, (2021).
25. Baldwin, J. R., Reuben, A., Newbury, J. B. & Danese, A. Agreement Between Prospective and Retrospective Measures of Childhood Maltreatment: A Systematic Review and Meta-analysis. *JAMA Psychiatry* **76**, 584–593 (2019).
26. Veer, I. M. *et al.* Psycho-social factors associated with mental resilience in the Corona lockdown. *Transl. Psychiatry* **11**, 1–11 (2021).
27. González-García, N. *et al.* Resilient functioning is associated with altered structural brain network topology in adolescents exposed to childhood adversity. *Dev. Psychopathol.* 1–11 (2023) doi:10.1017/S0954579423000901.
28. Eaton, S., Cornwell, H., Hamilton-Giachritsis, C. & Fairchild, G. Resilience and young people's brain structure, function and connectivity: A systematic review. *Neurosci. Biobehav. Rev.* **132**, 936–956 (2022).
29. Larsen, B., Sydnor, V. J., Keller, A. S., Yeo, B. T. T. & Satterthwaite, T. D. A critical period plasticity framework for the sensorimotor–association axis of cortical neurodevelopment. *Trends Neurosci.* **0**, (2023).
30. Luciana, M. & Collins, P. F. Neuroplasticity, the Prefrontal Cortex, and Psychopathology-Related Deviations in Cognitive Control. *Annul. Rev. Clin. Psychol.* **8** (2022)
31. Paus, T., Keshavan, M. & Giedd, J. N. Why do many psychiatric disorders emerge during adolescence? *Nat. Rev. Neurosci.* **9**, 947–957 (2008).
32. Sydnor, V. J. *et al.* Neurodevelopment of the association cortices: Patterns, mechanisms, and implications for psychopathology. *Neuron* **109**, 2820–2846 (2021).
33. Kiddle, B. *et al.* Cohort Profile: The NSPN 2400 Cohort: a developmental sample supporting the Wellcome Trust NeuroScience in Psychiatry Network. *Int. J. Epidemiol.* **47**, 18–19g (2018).
34. Coifman, R. R. & Lafon, S. Diffusion maps. *Appl. Comput. Harmon. Anal.* **21**, 5–30 (2006).
35. St Clair, M. C. *et al.* Characterising the latent structure and organisation of self-reported thoughts, feelings and behaviours in adolescents and young adults. *PLOS ONE* **12**, e0175381 (2017).
36. Bamber, D., Tamplin, A., Park, R. J., Kyte, Z. A. & Goodyer, I. M. Development of a short leyton obsessional inventory for children and adolescents. *J. Am. Acad. Child Adolesc. Psychiatry* **41**, 1246–1252 (2002).
37. Reynolds, C. R. & Richmond, B. O. What I think and feel: a revised measure of children's manifest anxiety. *J. Abnorm. Child Psychol.* **6**, 271–280 (1978).
38. Rosenberg, M. The measurement of self-esteem, Society and the adolescent self-image. *Princeton* 16–36 (1965).
39. Tennant, R. *et al.* The Warwick-Edinburgh Mental Well-being Scale (WEMWBS): development and UK validation. *Health Qual. Life Outcomes* **5**, 63 (2007).
40. Raine, A. The SPQ: a scale for the assessment of schizotypal personality based on DSM-III-R criteria. *Schizophr. Bull.* **17**, 555–564 (1991).
41. Horwood, J. *et al.* IQ and non-clinical psychotic symptoms in 12-year-olds: results from the ALSPAC birth cohort. *Br. J. Psychiatry J. Ment. Sci.* **193**, 185–191 (2008).
42. Goodyer, I. M., Herbert, J., Tamplin, A. & Altham, P. M. Recent life events, cortisol, dehydroepiandrosterone and the onset of major depression in high-risk adolescents. *Br. J. Psychiatry J. Ment. Sci.* **177**, 499–504 (2000).
43. Bernstein, D. P. *et al.* Development and validation of a brief screening version of the Childhood Trauma Questionnaire. *Child Abuse Negl.* **27**, 169–190 (2003).

44. Elgar, F. J., Waschbusch, D. A., Dadds, M. R. & Sigvaldason, N. Development and Validation of a Short Form of the Alabama Parenting Questionnaire. *J. Child Fam. Stud.* **16**, 243–259 (2007).
45. Parker, G. *et al.* The development of a refined measure of dysfunctional parenting and assessment of its relevance in patients with affective disorders. *Psychol. Med.* **27**, 1193–1203 (1997).
