# Supplementary material for: Longitudinal variation in resilient psychosocial functioning is associated with ongoing cortical myelination and functional reorganization during adolescence
Source: Nat Commun. 2024 Jul 29;15:6283. doi: 10.1038/s41467-024-50292-2 (PMC11286871; doi:10.1038/s41467-024-50292-2)
Supplement: Supplementary file 3 — Reporting Summary [file 41467_2024_50292_MOESM3_ESM.pdf]

nature portfolio

Corresponding author(s): Meike D Hettwer & Sofie L Valk

Last updated by author(s): Jun 6, 2024

Reporting Summary

Nature Portfolio wishes to improve the reproducibility of the work that we publish. This form provides structure for consistency and transparency in reporting. For further information on Nature Portfolio policies, see our Editorial Policies and the Editorial Policy Checklist.

Statistics

For all statistical analyses, confirm that the following items are present in the figure legend, table legend, main text, or Methods section.

n/a	Confirmed	
☐	☒	The exact sample size (*n*) for each experimental group/condition, given as a discrete number and unit of measurement
☐	☒	A statement on whether measurements were taken from distinct samples or whether the same sample was measured repeatedly
☐	☒	The statistical test(s) used AND whether they are one- or two-sided<br>*Only common tests should be described solely by name; describe more complex techniques in the Methods section.*
☐	☒	A description of all covariates tested
☐	☒	A description of any assumptions or corrections, such as tests of normality and adjustment for multiple comparisons
☐	☒	A full description of the statistical parameters including central tendency (e.g. means) or other basic estimates (e.g. regression coefficient) AND variation (e.g. standard deviation) or associated estimates of uncertainty (e.g. confidence intervals)
☐	☒	For null hypothesis testing, the test statistic (e.g. *F*, *t*, *r*) with confidence intervals, effect sizes, degrees of freedom and *P* value noted<br>*Give P values as exact values whenever suitable.*
☒	☐	For Bayesian analysis, information on the choice of priors and Markov chain Monte Carlo settings
☒	☐	For hierarchical and complex designs, identification of the appropriate level for tests and full reporting of outcomes
☐	☒	Estimates of effect sizes (e.g. Cohen's *d*, Pearson's *r*), indicating how they were calculated

Our web collection on statistics for biologists contains articles on many of the points above.

Software and code

Policy information about availability of computer code

Data collection	Data for each participant was recorded/transferred using the Research Electronic Data Capture (REDCap) software. Data was stored on Microsoft Access-based Cohort Management System (CMS).
Data analysis	Custom code generated for this project was made publicly available under https://github.com/CNG-LAB/cngopen/tree/main/adolescent_resilience/ScrFun and https://zenodo.org/records/11486553. Our analysis code makes use of open software: Gradient mapping analyses were carried out using BrainSpace (v. 0.1.2; https://brainspace.readthedocs.io/en/latest/) and surface visualizations were based on code from the ENIGMA Toolbox (v.1.1.3; https://enigma-toolbox.readthedocs.io/en/latest/; 117) in combination with ColorBrewer (v. 1.0.0; https://github.com/scottclowe/cbrewer2), and the Violin Plot (Holger Hoffmann (2024); https://www.mathworks.com/matlabcentral/fileexchange/45134-violin-plot). Statistical analyses were carried out using SurfStat (https://www.math.mcgill.ca/keith/surfstat/). Equivolumetric surfaces were computed using code from: https://github.com/MICA-MNI/micaopen/tree/master/a_moment_of_change 16. Z-tests were performed using the compare correlation coefficients function (Sisi Ma (2024). compare_correlation_coefficients (https://www.mathworks.com/matlabcentral/fileexchange/44658-compare_correlation_coefficients). Python: We made use of the following packages: scipy 1.10.1, sklearn 0.0.post1, matplotlib 3.7.1, numpy 1.24.2, pandas 1.5.3, seaborn 0.11.0.

For manuscripts utilizing custom algorithms or software that are central to the research but not yet described in published literature, software must be made available to editors and reviewers. We strongly encourage code deposition in a community repository (e.g. GitHub). See the Nature Portfolio guidelines for submitting code & software for further information.

Data

Policy information about availability of data

All manuscripts must include a data availability statement. This statement should provide the following information, where applicable:

- Accession codes, unique identifiers, or web links for publicly available datasets
- A description of any restrictions on data availability
- For clinical datasets or third party data, please ensure that the statement adheres to our policy

The behavioral resilience scores and microstructural profiles generated in this study have been deposited on Github and Zenodo under https://github.com/CNG-LAB/cngopen/tree/main/adolescent_resilience/ScrFun and https://zenodo.org/records/11486553. The item-level questionnaire data as well as unprocessed imaging data can be obtained from https://portal.ide-cam.org.uk/overview/6/managed or https://www.repository.cam.ac.uk/handle/1810/264350. The processed functional connectivity data are available at https://zenodo.org/records/6390852. The depicted data generated in this study are provided in the Supplementary Information/Source Data file.

Research involving human participants, their data, or biological material

Policy information about studies with human participants or human data. See also policy information about sex, gender (identity/presentation), and sexual orientation and race, ethnicity and racism.

Reporting on sex and gender	Participants self-identified as either male or female in the 'sex' variable of the questionnaire. It was not clarified if some understood the question as 'gender identity', socially attributed or biological category. Thus, data on divergence between sex and gender was not available for this study. Self-reported sex was included as a covariate in all general linear models.
Reporting on race, ethnicity, or other socially relevant groupings	We indicate data on race/ethnicity in the demographics overview but did not include this information in statistical analyses. This is mainly because we had no theory-driven hypothesis that race would influence the association between resilience trajectories and myeloarchitectural maturation. The majority of individuals (1764 out of 2245) indicated 'White' as their ethnicity.
Population characteristics	The full NSPN sample included in this study comprised 2245 individuals (mean age at baseline: 19.06 +/- 3.02 years; 1210 female). We computed a psychosocial distress score in n=1533 individuals (mean age at baseline: 19.25 +/- 3.02 years; 845 females), derived psychosocial resilience scores in n=712 individuals (mean age at baseline = 18.65 +/- 3 years; 365 females), and finally assessed the association between changes in psychosocial resilience and brain maturation in n = 141 individuals (mean age at baseline = 18.44 +/- 2.90, 71 female).
Recruitment	Source: https://academic.oup.com/ije/article/47/1/18/4644452#118180486: "The NIHR Primary Care Research Network (PCRN) engaged 50 GP's to recruit young people using their sex-age registers by sending out invitations (including an expressions of interest form (EoI)) across Cambridgeshire and Greater London (closest proximity to universities leading the study). Schools and Further Education colleges were also engaged to distribute the EoI forms to 14 to 18-year-old participants. The NSPN recruitment team assisted GP's and schools by providing invitation to participate letters, which were forwarded to potential participant's home address that remained unknown to the NSPN investigators. Purposive advertisement was also used during recruitment; invitation letters with EoI were sent to those who responded to advertisements that met the age criteria. If an individual wanted to participate they informed NSPN recruitment team over the phone/sent in completed EoI form."<br><br>A possible bias is that the study requires substantial time committment from participants and may thus filter out adolescents (and parents) who are willing to commit to that. This bias has been reported to be reflected in higher parental educational attainment when compared with the general population, which may in turn impact measures of adversity exposure included for this study. However, as this bias applies to all included participants and inter-individual comparisons have only been made within the cohort, we assume the impact of this bias on current conclusions is minor.
Ethics oversight	This study was conducted in accordance with U.K. National Health Service research governance standards and participants provided informed written consent during NSPN data acquisition, for which ethical approval was granted by the Cambridge East Research Ethics Committee under REC 12/EE/0250.

Note that full information on the approval of the study protocol must also be provided in the manuscript.

Field-specific reporting

Please select the one below that is the best fit for your research. If you are not sure, read the appropriate sections before making your selection.

☐ Life sciences ☒ Behavioural & social sciences ☐ Ecological, evolutionary & environmental sciences

For a reference copy of the document with all sections, see nature.com/documents/nr-reporting-summary-flat.pdf

Behavioural & social sciences study design

All studies must disclose on these points even when the disclosure is negative.

Study description	The study in part includes quantitative data (also subjective assessments are recorded as numbers on scales) and quantitative statistical analyses such as general linear models and linear mixed-effects models.
Research sample	The sample comprises adolescents and young adults at ages 14-24 (baseline) from the Greater London and Cambridgeshire area. Demographics for all participants included in either behavioral or neuroimaging sub-abnalyses: Total (N = 2245)<br><br>Age at baseline: 19.06 ± 3.02y<br>n per age bin (14-15, 16-17, 18-19, 20-21, >22): 446/483/432/421/462<br>Sex: 1035 male / 1210 female<br>HPQ Sites: UCL: 947, Cambridge: 1298<br>IMD: 15.17 (± 11.80)<br>Ethnicity: Asian / Asian British: 201; Black/ African / Caribbean / Black British: 91; Multiple ethnic groups: 135; White: 1764; Other: 32; Unknown: 22<br><br>The NSPN cohort is a publicly available dataset that is well phenotypes. We selected it for the current study as it comprises both 1) multi-modal repeated imaging as well as 2) longitudinal behavioral assessments including both risk factor exposure and mental health variables.
Sampling strategy	The NSPN 2400 Cohort (all individuals, including those with behavioral but no imaging data) aimed to recruit at least 2000 participants in an age- sex-stratified sample from the general population (Greater London and Cambridgeshire), including equal numbers of males and females for the following five age groups: 14-15, 16-17, 18-19, 20-21, and 22-24.99years. Individuals of the MRI cohort were recruited within the ten age-sex strata. The current study includes all 2245 individuals for behavioral analyses, but only 141 individuals for the testing of associations between changes in psychosocial resilience and brain maturation. This final sample size was defined by data availability, restricting analyses to only individuals for which both longitudinal neuroimaging and longitudinal behavioral risk assessment was present.
Data collection	Extensive questionnaires were filled out at home at the computer (Home questionnaire packs), with no researcher present. There were no experimental conditions to be aware of by either the participant or researchers involved. MRI data was collected at the scanner with researchers present, but again, no relevant experimental conditions or hypotheses as the dataset was initially collected as a general populatin / descriptive dataset.
Timing	Participants were recruited starting 2012 until 2017. Participants were followed-up for one to two years (on average ~1.25 years between measurement time-points)
Data exclusions	From originally 295 subjects with imaging data, 154 were excluded because they did not have repeated imaging and behavioral data (this includes single questionnaires being incomplete), or because scans were of low quality (36 scans: 17 scans were excluded due to high in-scanner motion (defined as mean FD > 0.3 mm or maximum FD > 1.3 mm), 9 due to coregistration errors, 7 due to a lack of convergence of the ME-ICA algorithm, 2 due to parcellation errors, and 1 due to extensive signal dropout.
Non-participation	Out of the total behavioral sample (N = 2245), n=318 individuals were invited for the MRI experiments. For n=141 individuals, longitudinal data was available for both neuroimaging and behavioral assessments.
Randomization	n/a, there were no experimental groups in the initial data collection.

Reporting for specific materials, systems and methods

We require information from authors about some types of materials, experimental systems and methods used in many studies. Here, indicate whether each material, system or method listed is relevant to your study. If you are not sure if a list item applies to your research, read the appropriate section before selecting a response.

Materials & experimental systems

n/a	Involved in the study	
☒	☐	Antibodies
☒	☐	Eukaryotic cell lines
☒	☐	Palaeontology and archaeology
☒	☐	Animals and other organisms
☒	☐	Clinical data
☒	☐	Dual use research of concern
☒	☐	Plants

Methods

n/a	Involved in the study	
☒	☐	ChIP-seq
☒	☐	Flow cytometry
☐	☒	MRI-based neuroimaging

Plants

Seed stocks

Report on the source of all seed stocks or other plant material used. If applicable, state the seed stock centre and catalogue number. If plant specimens were collected from the field, describe the collection location, date and sampling procedures.

Novel plant genotypes

Describe the methods by which all novel plant genotypes were produced. This includes those generated by transgenic approaches, gene editing, chemical/radiation-based mutagenesis and hybridization. For transgenic lines, describe the transformation method, the number of independent lines analyzed and the generation upon which experiments were performed. For gene-edited lines, describe the editor used, the endogenous sequence targeted for editing, the targeting guide RNA sequence (if applicable) and how the editor was applied.

Authentication

Describe any authentication procedures for each seed stock used or novel genotype generated. Describe any experiments used to assess the effect of a mutation and, where applicable, how potential secondary effects (e.g. second site T-DNA insertions, mosiacism, off-target gene editing) were examined.

Magnetic resonance imaging

Experimental design

Design type

fMRI: resting-state, otherwise structural data (T1 and Magnetic Transfer (MT))

Design specifications

none

Behavioral performance measures

none

Acquisition

Imaging type(s)

structural (T1 and MPM)

Field strength

3T

Sequence & imaging parameters

Myelin-sensitive MRI

MPM comprised three multi-echo 3D FLASH scans: predominant T1-weighting (repetition time (TR) = 18.7 ms, flip angle = 20°), and predominant proton density (PD) and MT-weighting (TR= 23.7 ms; flip angle = 6°). To achieve MT-weighting, an off-resonance Gaussian-shaped RF pulse (duration = 4 ms, nominal flip angle = 220̊, frequency offset from water resonance = 2 kHz) was applied prior to the excitation. For MT weighted acquisition, several gradient echoes were recorded with alternate readout polarity at six equidistant echo durations (TE) between 2.2 and 14.7 ms. The longitudinal relaxation rate and MT signal are separated by the MT saturation parameter, creating a semi-quantitative measurement that is resistant to field inhomogeneities and relaxation times 22,88. Further acquisition parameters: 1 mm isotropic resolution, 176 sagittal partitions, field of view (FOV) = 256×240 mm, matrix = 256×240×176, parallel imaging using GRAPPA factor two in phase-encoding (PE) direction (AP), 6/8 partial Fourier in partition direction, non-selective RF excitation, readout bandwidth BW = 425 Hz/pixel, RF spoiling phase increment = 50̊.

Resting-state functional MRI

Resting-state functional MRI (fMRI) data were acquired using a multiecho echo-planar imaging sequence (TR = 2.42 s; GRAPPA with acceleration factor = 2; flip angle = 90°; matrix size = 64×64×34; FOV = 240 × 240 mm; in plane resolution = 3.75 mm×3.75 mm; slice thickness = 3.75 mm with 10% gap, sequential slice acquisition, 34 oblique slices; bandwidth, 2368 Hz/pixel; TE = 13, 30.55, and 48.1 ms.

Area of acquisition

whole brain scan

Diffusion MRI ☐ Used ☒ Not used

Preprocessing

Preprocessing software

Structural: Standard FreeSurfer recon-all pipeline. Freesurfer v5.3.0 was used to process individual structural scans with a pipeline including, amongst others, skull-stripping, segmentation of cortical grey and white matter and reconstruction of the cortical surface and grey-white matter boundary.

Functional : AFNI was used for basic preprocessing of functional MRI scans. All volumes acquired during steady-state equilibration (15 s) were discarded. Motion correction parameters and parameters for anatomical-functional coregistration were calculated from the images acquired with TE = 30.55 ms. The first volume after equilibration was used as the base EPI image. Matrices for de-obliquing and six-parameter rigid body motion correction were computed. Then, 12-parameter affine anatomical-functional coregistration was computed using the LPC cost function (96), with the EPI base image as the LPC weight mask. Matrices for de-obliquing, motion correction, and anatomical-functional coregistration were combined into a single alignment matrix using the concatenation approach from the AFNI tool alignepianat.py. The images for each TE were then

slice-time corrected and spatially aligned through application of the alignment matrix. The coregistration of structural and functional scans was visually assessed.

Normalization

Structural: FreeSurfers non-linear transform to template.
Functional: 12-parameter affine anatomical-functional coregistration was computed using the LPC cost function.

Normalization template

MNI152 template in Talairach space

Noise and artifact removal

Functional MRI data were preprocessed using multi-echo independent component analysis (MEICA). MEICA identifies and removes sources of variance in the times series that do not scale linearly with TE and are therefore assumed not to be representative of the BOLD signal. The retained independent components, representing BOLD contrast, were optimally recomposed to generate a broadband denoised fMRI time series at each voxel. Regional time series were averaged over all voxels within each parcel and bandpass filtered by the discrete wavelet transform, corresponding to a frequency range of 0.025-0.111 Hz.

Volume censoring

An overall estimate of head motion by each participant, mean framewise displacement (FD), was calculated from the six motion parameter time series (three rotation and three translation parameters) estimated during scan re-alignment. Individual functional connectivity matrices were estimated by Pearson's correlation for each possible of pair of nodes. Here, we regressed each pairwise correlation or edge on the time-averaged head motion of each participant (mean FD). The residuals of this regression were the estimates of functional connectivity used for further analysis.

Statistical modeling & inference

Model type and settings

Brain-behavior associations were assessed via mass-univariate analyses, including the independent variable (here change in stressor resilience scores) as well as covariates (age, sex, site) as fixed effects. In analyses assessing age effects via linear mixed-effect models as part of the maturational index computation, SubjectIDs were included as random effect to account for repeated measures.

Effect(s) tested

Most described analyses test continuous associations via general linear models (GLMs) / regressions. Group differences in the Maturational Index were computed via Fisher-z-test.

Specify type of analysis: ☐ Whole brain ☐ ROI-based ☒ Both

Anatomical location(s)

All analyses are based on parcellated data, yielding 360 parcels /ROIs for structural analyses. For one analysis, namely the functional contextualization of structural effects in the anterolateral prefrontal cortex, we define all parcels that show a structural effect as a ROI, or seed, for a seed-based functional connectivity annotation.

Statistic type for inference

(See Eklund et al. 2016)

We did not use voxel-level data.

Correction

We applied both non-parametric permutation testing (10.000 permutations) and FDR corrections.

Models & analysis

n/a	Involved in the study
☐	☒ Functional and/or effective connectivity
☒	☐ Graph analysis
☐	☒ Multivariate modeling or predictive analysis

Functional and/or effective connectivity

Pearson correlation

Multivariate modeling and predictive analysis

For the prediction of distress scores to derive stressor resilience scores:
Independent variables included summary scores from the Life Events Questionnaire (LEQ), Child Trauma Questionnaire (CTQ), Alabama Parenting Questionnaire (APQ), Measure of Parenting Style (MOPS), and socioeconomic status (as approximated by zip codes/IMD).

We used a random forest regression in a supervised machine learning approach implemented in sci-kit learn (v1.2.1, https://scikit-learn.org, in Python v3.10.9). We applied a nested cross-validation in which we left all sessions of one subject out in the outer scheme, i.e., 712 outer folds, and split the remaining data into 5 even groups for training, i.e., 5 inner folds, in each iteration. Performance was estimated based on mean absolute errors and parameter optimization was performed for the number of estimators (50, 100, 150, 200, 250, 300) and tree depth (5 to 15). We included a StandardScaler (z-scoring) to preprocess features within the cross-validation scheme.
